# Participation and Functional Independence in Adults With Recessive Spastic Ataxia of Charlevoix-Saguenay

**DOI:** 10.1177/00084174221088417

**Published:** 2022-04-26

**Authors:** Samar Muslemani, Isabelle Lessard, Caroline Lavoie, Isabelle Côté, Bernard Brais, Jean Mathieu, Cynthia Gagnon

**Keywords:** Activities of daily living, Recessive spastic ataxia, Social participation, Activités de la vie quotidienne, Ataxie récessive spastique, Participation sociale‌

## Abstract

**Background.** Autosomal recessive spastic ataxia of Charlevoix-Saguenay (ARSACS) is a progressive disorder where upper and lower extremities motor performances may bring participation restriction. **Purpose.** To document participation in adults with ARSAC and explore associations with motor performances. **Method.** Twenty-eight participants took part in the study. Participation was assessed using the LIFE-H. Motor performance was assessed using several outcomes including the SARA, LEMOCOT, Berg Balance Scale, 10-Meter Walk Test, and Finger-to-nose Test. **Findings.** Participation was significantly lower in the wheelchair user subgroup. Also, for 29 activities out of 77, at least 15% of participants reported severely disrupted participation. Participation was correlated with upper and lower limbs coordination, walking ability, balance, disease severity, and fine dexterity (Spearman *r* = .41–0.85, *p* < .03). **Implications.** Results showed significant participation restrictions and suggest that interventions aiming to improve or compensate upper and lower limbs functions could help to decrease disease burden.

## Résumé

**Description.** L’ataxie récessive spastique de Charlevoix-Saguenay (ARSCS) est une maladie évolutive qui affecte la motricité des membres supérieurs et inférieurs et peut restreindre la participation aux activités. **But.** Documenter la participation chez les personnes adultes atteintes de l’ARSCS et explorer les associations avec les capacités motrices. **Méthodologie.** Vingt-huit personnes ont participé à l’étude. La participation a été évaluée à l’aide de la MHAVIE. Les capacités motrices ont été évaluées notamment à l’aide du SARA, du LEMOCOT, de l’échelle d’évaluation de l’équilibre de Berg, du test de marche de 10 mètres et de l’épreuve doigt-nez. **Résultats.** La participation était significativement plus faible dans le sous-groupe des personnes en fauteuil roulant. Également, pour 29 activités sur 75, au moins 15% des participants ont déclaré que leur participation était gravement perturbée. La participation était corrélée à la coordination des membres supérieurs et inférieurs, à la capacité de marche, à l’équilibre, à la gravité de la maladie, et à la dextérité fine (*r* de Spearman = 0,41–0,85, *p* < .03). **Conséquences.** Les résultats démontrent des restrictions importantes à la participation et suggèrent que des interventions visant à améliorer ou à compenser les fonctions des membres supérieurs et inférieurs pourraient aider à réduire le fardeau de la maladie.

## Introduction

Autosomal recessive spastic ataxia of Charlevoix-Saguenay (ARSACS) is a progressive hereditary movement disorder (Bouchard et al., 1998; Engert et al., 2000) and is one of the most frequent recessive ataxia after Friedreich's ataxia (Synofzik et al., 2013), with the highest prevalence worldwide found in Québec, Canada (De Braekeleer et al., 1993). It most often presents as a pediatric-onset disease with variable involvement of the pyramidal, cerebellar and neuropathic structures, thereby leading to a variety of clinical phenotypes (an individual's observable traits) (Bouchard et al., 1998; Gagnon et al., 2018a). Decreased motor performance is a key feature of ARSACS, but also includes upper and lower limb incoordination, impaired balance and walking difficulties (Briand et al., 2019; Duquette et al., 2013; Gagnon et al., 2018a, 2018b). Mobility level varies significantly from one person to another: for example, indoor wheelchair use is reported in 45% of affected individuals, with an age of wheelchair permanent use (for both indoor and outdoor activities) ranging from 19 to 48 years old (Gagnon et al., 2018a). Upper extremity motor functions, including coordination, dexterity and prehension, are affected early in the course of the disease (Gagnon, Desrosiers, et al., 2004). Motor performance was associated with significant participation restrictions in ARSACS (Gagnon, Desrosiers, et al., 2004). Participation refers to the accomplishment and engagement in meaningful daily and social activities. Participation restriction is defined by the Human Development Model-Disability Creation Process (HDM-DCP) framework as difficulty and/or assistance requirement to accomplish daily and social activities (Fougeyrollas, 2010). To guide policy development and service delivery, the HDM-DCP conceptualised participation restriction as a result of a disruptive interaction over time of personal and environmental factors (Fougeyrollas et al., 2019). To our knowledge, only two studies both conducted by our group documented the level of participation in daily and social activities, where older adults (≥40 years) showed lower global participation level compared to younger ones (<40 years) (Gagnon et al., 2018a; Gagnon, Desrosiers, et al., 2004). However, their satisfaction associated with their level of participation in different activity domains is unknown. Since satisfaction is shown to be strongly associated with quality of life in other populations (Levasseur et al., 2004), this gap should be filled to better manage participation restrictions in this population. Also, participation restriction could be a predictor of a reduced cognitive functioning (Bourassa et al., 2017) and greater risk of mortality in aging population (Holt-Lunstad et al., 2010).

A better understanding of the participation level of people with ARSACS in different domains such as mobility, work and leisure, in addition to the identification of motor impairments associated with participation restrictions, would allow to identify specific areas where occupational therapists could intervene to help these individuals and, ultimately, to develop better anticipatory guidance. A prognostic approach could be put in place by providing in advance healthcare and community services to compensate for upcoming loss of motor functions (Raymond et al., 2019). The objectives of the present study were thus to, in adults with ARSACS: (1) document participation level in daily and social activities; (2) compare participation level across sex and indoor mobility levels; and (3) explore the associations between participation level and motor performances and participation satisfaction level.

## Method

### Study Design

An exploratory cross-sectional study was used to describe participation.

### Participants

Participants were recruited among a subset of 175 patients with ARSACS followed at the Neuromuscular Clinic of the *Centre Intégré Universitaire de Santé et de Services Sociaux* (CIUSSS) *du Saguenay–Lac-St-Jean* (Quebec, Canada), also described in Lessard et al. (2017) and Gagnon et al. (2018a). Inclusion criteria were: (1) ≥ 18 year-old, (2) genetically confirmed diagnosis of ARSACS homozygous for the *SACS* c.8844delT mutation, and (3) able to provide informed consent. Patients were excluded if they lived outside 50 km from the clinic, had other pathologies causing functional limitations, a Baclofen intrathecal pump or were pregnant. The study was approved by the Ethics Review Board of the *CIUSSS du Saguenay–Lac-St-Jean* (#2012-012; approved 2012, September 21). A written informed consent was obtained from all participants.

### Data Collection

This study was part of a larger data collection that aimed to document the natural history of the disease. In this larger study, participants were seen over three half-day sessions within a 2-week period. Each session was equivalent in terms of duration and difficulty level. The Berg Balance Scale (BBS), the Lower Extremity Motor Coordination Test (LEMOCOT), the Purdue Pegboard Test (PPT), and the Nine Hole Peg Test (NHPT) were administered during the first visit; the LIFE-H, the Scale for the Assessment and Rating of Ataxia (SARA), and the Standardized Finger-to-nose Test (SFNT) were administered during the second visit; while the Barthel Index, the Six-Minute Walk Test (6MWT), and the 10-Meter Walk Test (10mWT) were administered during the third visit. All these measures were administered by two trained research physical therapists using standardized operational procedures. A short questionnaire was also answered by participants to obtain information about their age, sex, indoor mobility level, use of walking aids and employment status. The two questions about indoor mobility and walking aid use served to classify participants based on their mobility level (according to stages defined in the SARA development study (Schmitz-Hubsch et al., 2006)): (Group 1) First walking difficulty, no walking aid; (Group 2) Walk with aid or support; and (Group 3) Wheelchair user. First walking difficulty corresponds to individuals with mild imbalance and alteration of walking gait, not enough to require the use of a walking aid.

### Measures

#### Participation measures

The LIFE-H questionnaire (short version 3.1) was used to assess participation (Fougeyrollas et al., 1998). It consists of 77 items, or activities, which cover the 12 domains of participation (number of activities): nutrition (4), fitness (4), personal care (8), communication (8), housing (8), mobility (5), responsibilities (8), interpersonal relationships (7), community life (8), education (2), work (8), and leisure (7). The first six domains address participation in activities of daily living (ADLs) whereas the others refer to participation in social activities (formally known as social roles). According to administration procedures, there is a score for each domain, two sub-scores (daily and social activities) and a total score (global participation). Considering that few participants were in study during the data collection, the education domain was not included in the total score. The LIFE-H measures both accomplishment and satisfaction levels in participation. Accomplishment includes difficulty and assistance required: a mean score of “0” refers to a complete disruption of participation (i.e., activity is not accomplished), and “9” refers to full participation (i.e., activity is accomplished without difficulty and assistance). A change of 0.5 (/9) is considered a minimal clinically important difference (Desrosiers et al., 2006). A score of five or less indicates a *severely disrupted participation* (i.e., activities not accomplished or accomplished with human assistance) (Fougeyrollas et al., 2003). The satisfaction level is measured on a 5-point scale, where a higher score indicates a greater satisfaction. The LIFE-H was scored by the therapist after asking questions to the participants through a clinical interview. In addition, the Barthel Index (Mahoney & Barthel, 1965) was also used to measure independence level in ADLs and mobility as it is often used in other ataxias and will allow comparison across different ataxias. The index is an ordinal scale that includes 10 items, each relating to a specific activity: feeding, bathing, grooming, dressing, bowels, bladder, toilet use, transfers, mobility and stairs. It can be used with any clientele with a functional loss of independence. Most items have a maximum score of 10 points, scoring 0 for inability to perform the task, 5 points if any assistance is required, and 10 for independence (Shah et al., 1989). A total score of 0 to 20 suggests total dependence, 21–60 severe dependence, 61–90 moderate dependence, and 91–99 slight dependence. The maximum score is 100 points and represents total independence. The Barthel Index demonstrated high inter-rater reliability (ICC = 0.95) and test–retest reliability (ICC = 0.89) as well as high correlations (*r = *0.74–0.8) with other measures of physical disability in head-injury and stroke populations (Collin et al., 1988).

#### Motor performances outcome measures

Ataxia severity was quantified using the SARA (Schmitz-Hubsch et al., 2006), a generic scale designed to assess cerebellar ataxia scored by the assessor. It includes eight items related to mobility, balance, speech and coordination, for a total score ranging from 0 (no ataxia) to 40 (most severe ataxia). Lower limb coordination was assessed using the LEMOCOT (Desrosiers, Rochette, et al., 2005; Lessard et al., 2017). Sitting down, participants alternatively touch with their foot a proximal and a distal target placed 30 cm from each other as fast as possible for 20 s. One trial for each side was done. The number of touched targets is recorded, a higher score suggesting a better lower limb coordination. Balance was assessed using the BBS (Berg et al., 1989; Lessard et al., 2018). It contains 14 items graded from 0 to 4, for a maximum score of 56 (higher values indicate better performance). Items vary in terms of difficulty, ranging from maintaining a seated position to a one-leg stand. Walking ability was measured using two indicators: (1) short distance walking speed, assessed using the 10mWT, and (2) long distance walking capacity, using the 6MWT. During the 10mWT (Lessard et al., 2018), participants walk at their own comfortable speed on a 10 m distance. The time in seconds to complete the task was recorded and the speed in m/s was then calculated and used for analysis. In the 6MWT (Guyatt et al., 1985; Lessard et al., 2018), participants walk along a 30 m corridor as fast as possible to cover the longest distance possible. The total distance in meters covered in a 6 min period was recorded. Fine dexterity was assessed using the PPT and NHPT. In the PPT (Tiffin, 1968), the number of pegs placed on the board by the dominant hand during a 30 s period was counted (mean of two trials). For the NHPT (Mathiowetz et al., 1985), participants had to insert and remove nine pegs from holes on a board as quickly as possible, and the time to complete the task in seconds was recorded. The mean of two trials was used. Finally, the SFNT (Desrosiers et al., 1995) was used to measure upper extremity motor coordination. With their index finger, participants moved horizontally from their nose to a target placed 45 cm away at eye level as quickly as possible for 20 s. The mean of two trials was used. Convergent validity of the SFNT was demonstrated in ARSACS by moderate to strong correlations with gross and fine finger dexterity (*r* = 0.82–0.84), global upper extremity performance (0.74–0.79), functional independence (*r* = 0.74) and social participation (*r* = 0.78) (Gagnon, Mathieu, et al., 2004). For all measures regarding the upper arm functions (PPT, NHPT, and SFNT), scores of the dominant side were used in the data analysis.

## Data Analysis

Descriptive statistics are presented as the median [minimum–maximum] for continuous variables, and as frequency (%) for categorical variables. Distribution normality of the total score for the LIFE-H and Barthel Index was assessed using the Kolmogorov–Smirnov test. Characteristics of the population include age, sex, and employment status, and are presented for the total sample and for each mobility level. Comparisons were made using the Kruskal–Wallis test or the Mann–Whitney *U* test. The LIFE-H (total score, each domain, two subscores and satisfaction) and Barthel Index (total score and items) were compared between participants according to their mobility level using the Kruskal–Wallis test. The LIFE-H (total score, each domain and two subscores) and Barthel Index (total score) were compared between sexes using the Mann–Whitney *U* test. For each activity of the LIFE-H, participants were categorized according to their level of social participation: adequate participation (score > 5); severely disrupted participation (score ≤ 5); or not applicable because of the participant's specific context. Spearman correlation coefficient was used to document the association of participation (LIFE-H total score and two subscores) and independence (Barthel Index total score) with motor performance outcome measures. The association between satisfaction and participation level was also documented using Spearman correlation coefficient. The following criteria were used as a general guideline to define correlation coefficients: <0.25—little or no relationship; 0.25–0.50—fair relationship; 0.50–0.75—moderate to good relationship; above 0.75 good to excellent relationship (Portney & Watkins, 2000). For all tests, a *p*-value < .05 is considered significant. Data were analyzed using IBM SPSS Statistics for Windows, Version 24.0 (Armonk, NY: IBM Corp).

## Findings

### Participants’ Characteristics

As described in Lessard et al., 116 individuals out of the 175 followed at the neuromuscular clinic were not eligible for the larger study due to heterozygosity for the 8844delT mutation, age range or being outside the 50 km around the clinic (Lessard et al., 2017). From the 59 remaining eligible individuals, 15 persons refused to participate, 14 could not be contacted during the study timeframe, and two persons abandoned before the end of the study (Lessard et al., 2017). A total of 28 people participated in the study with a mean age of 38.1 years. Characteristics of each mobility level subgroup are presented in Table 1. Subgroups are not different in terms of sex and employment status, but age increases significantly as the need for walking aid increases.

**Table 1.** Characteristics of the Study Population

Characteristic	Total group (n = 28)	Mobility level			*p*-value
		No walking aid (n = 7)	Walking aid (n = 11)	Wheelchair (n = 10)	
Age (y)					
Mean (SD)	38.1 (12.6)	26.0 (5.7)	34.7 (9.1)	50.3 (8.5)	<.001
min–max	18–59	18–33	21–50	32–59	
Sex, *n* (%)					
Men	16 (57.1)	4 (57.1)	5 (45.5)	7 (70.0)	.525
Women	12 (42.9)	3 (42.9)	6 (54.5)	3 (30.0)	
Age—Permanent use of wheelchair, *(y)* (n = 10)						
Mean (SD)	38.9 (7.7)	--	--	38.9 (7.7)	
min-max	30-49	--	--	30-49	
Employment status, *n (%)*					
At work	4 (14.3)	2 (28.6)	1 (9.1)	1 (10.0)	.316
At school	4 (14.3)	2 (28.6)	2 (18.2)	0	
Not at work/school	19 (67.9)	3 (42.9)	8 (72.7)	8 (80.0)	
Missing information	1 (3.5)	--	--	1 (10.0)	

### Participation

**Figure 1.**
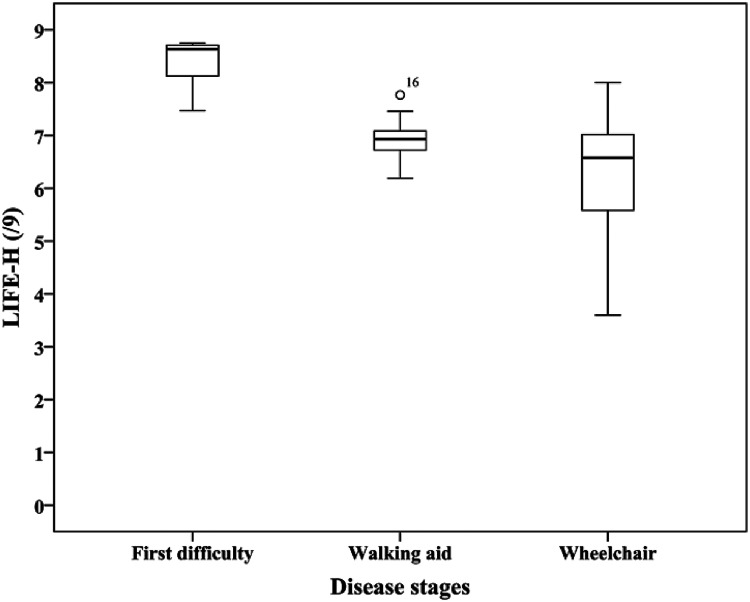
Score distribution of the LIFE-H for each mobility subgroups.

The LIFE-H total score is normally distributed (*p* = 0.2), and scores distribution for each subgroup is shown in Figure 1. The accomplishment level for each domain of the LIFE-H is reported in Table 2. No differences between men and women were found for all domains and subscores, except for Interpersonal relationships, where women had a slightly better participation score (median = 8.8 for women and 8.6 for men, *p* = .047). Results were also compared between the three subgroups of participants according to their mobility level, and a significant difference was found for most domains; overall, participants with first walking difficulties reported better participation than those using a wheelchair. No significant differences were found for the Responsibility, Interpersonal relationships, and Employment domains. For the subgroup with first walking difficulties, the total score was close to the maximum score (median score = 8.6 vs. max score = 9.0), which suggests few problems to accomplish the activities.

**Table 2.** Comparison of Scores for Each Domain, Subscores and Total Score for the LIFE-H Between the Three Subgroups of Participants

	Participation score^a^				*p* value
	Total	First diff. (n = 7)	Walking aid (n = 11)	Wheelchair (n = 10)	
*Daily activities domain*					
Nutrition	7.0 [2.5–9.0]^b^	8.5 [7.5–9.0]	7.0 [6.0–8.5]	6.0 [2.5–7.8]	.001
Fitness	6.8 [3.3–9.0]	9.0 [6.0–9.0]	6.5 [5.3–8.7]	6.3 [3.2–8.0]	.012
Personal care	8.0 [2.1–8.9]	8.8 [8.0–8.9]	8.0 [6.1–8.5]	5.7 [2.1–8.1]	<.001
Communication	8.6 [6.4–9.0]	9.0 [8.5–9.0]	8.6 [7.8–9.0]	8.0 [6.4–9.0]	.027
Housing	6.1 [3.1–9.0]	8.4 [6.3–9.0]	5.9 [4.1–7.1]	4.5 [3.1–6.8]	.001
Mobility	5.4 [1.7–9.0]	8.3 [5.2–9.0]	4.0 [2.0–7.2]	4.7 [1.7–7.3]	.003
*Subscore*	7.0 [3.7–8.9]	8.5 [7.1–8.9]	7.0 [6.3–7.8]	6.0 [3.7–7.6]	<.001
*Social activities domain*					
Responsibility	9.1 [4.5–9.0]	9.0 [9.0–9.0]	9.0 [7.8–9.0]	8.7 [4.5–9.0]	.066
Interpersonal relationships	8.8 [6.7–9.0]	8.8 [7.5–9.0]	8.8 [7.2–9.0]	8.8 [6.7–9.0]	.645
Community life	7.4 [3.3–9.0]	9.0 [7.3–9.0]	6.6 [4.8–8.4]	7.3 [3.3–8.7]	.008
Employment (n = 25)	6.5 [0–8.7]	7.5 [3.7–8.7]	6.0 [2.0–8.0]	6.3 [0–8.6]	.073
Recreation	4.5 [0–9.0]	8.6 [3.7–9.0]	4.2 [0–6.3]	3.5 [0–8.0]	.006
*Subscore*	7.3 [3.5–8.9]	8.5 [7.4–8.9]	6.9 [5.9–7.8]	7.3 [3.5–8.6]	.002
Total score	7.0 [3.6–8.8]	8.6 [7.5–8.8]	6.9 [6.2–7.8]	6.6 [3.6–8.0]	.001

^a^ Maximum score is 9.0; a higher score refers to higher participation level.

^b^ All results are expressed as Median [min–max].

A more in-depth data analysis was conducted to determine which items from each domain have at least five participants who reported a severely disrupted participation (Table 3). Housing, Mobility, Community, and Leisure were the most affected domains.

**Table 3:** Activities Where At least Five Participants Reported Severely Disrupted Participation

Domain	Item	Number of participants^a^		
		Disrupted	Adequate	N/A
Daily activities domain				
Nutrition	Preparing your meals	11	17	0
	Eating in restaurants	19	9	0
Fitness	Getting in and out of bed	5	23	0
	Participating in physical activities to maintain fitness	11	15	2
Personal care	Attending to your personal hygiene	5	23	0
	Using the bathroom and toilet in your home	5	23	0
	Dressing and undressing the lower half of your body	5	23	0
	Putting on, removing, and maintaining your assistive devices	8	17	3
Housing	Maintaining your home	19	9	0
	Maintaining the grounds of your home	16	6	6
	Doing major household tasks	20	5	3
	Moving around outside your home	6	22	0
Mobility	Getting around streets or sidewalk	9	19	0
	Getting around on slippery or uneven surfaces	13	15	0
	Riding a bicycle	13	9	6
	Being a passenger in a vehicle	6	21	1
Social activities domain				
Responsibility	Using bank cards and automatic teller machines	5	23	0
Community life	Getting to public buildings in your community	9	19	0
	Entering and getting around in public buildings in your community	9	19	0
	Getting to commercial establishments in your community	11	17	0
	Entering and moving around in commercial establishments in your community	13	15	0
Employment	Choosing a career or profession	5	13	10
	Seeking employment	5	5	17
	Holding a paid job	15	4	9
Leisure	Participating in sporting or recreational activities	8	17	3
	Going to sporting events	9	8	11
	Going to artistic or cultural events	11	13	4
	Participating in tourist activities	17	8	3
	Taking part in outdoor activities	12	10	6

^a^ Number of participants who reported a disrupted participation (score of 5 or less), an adequate participation (score over 5), or for whom the item was not applicable (N/A) because of their specific circumstances.

**Figure 2.**
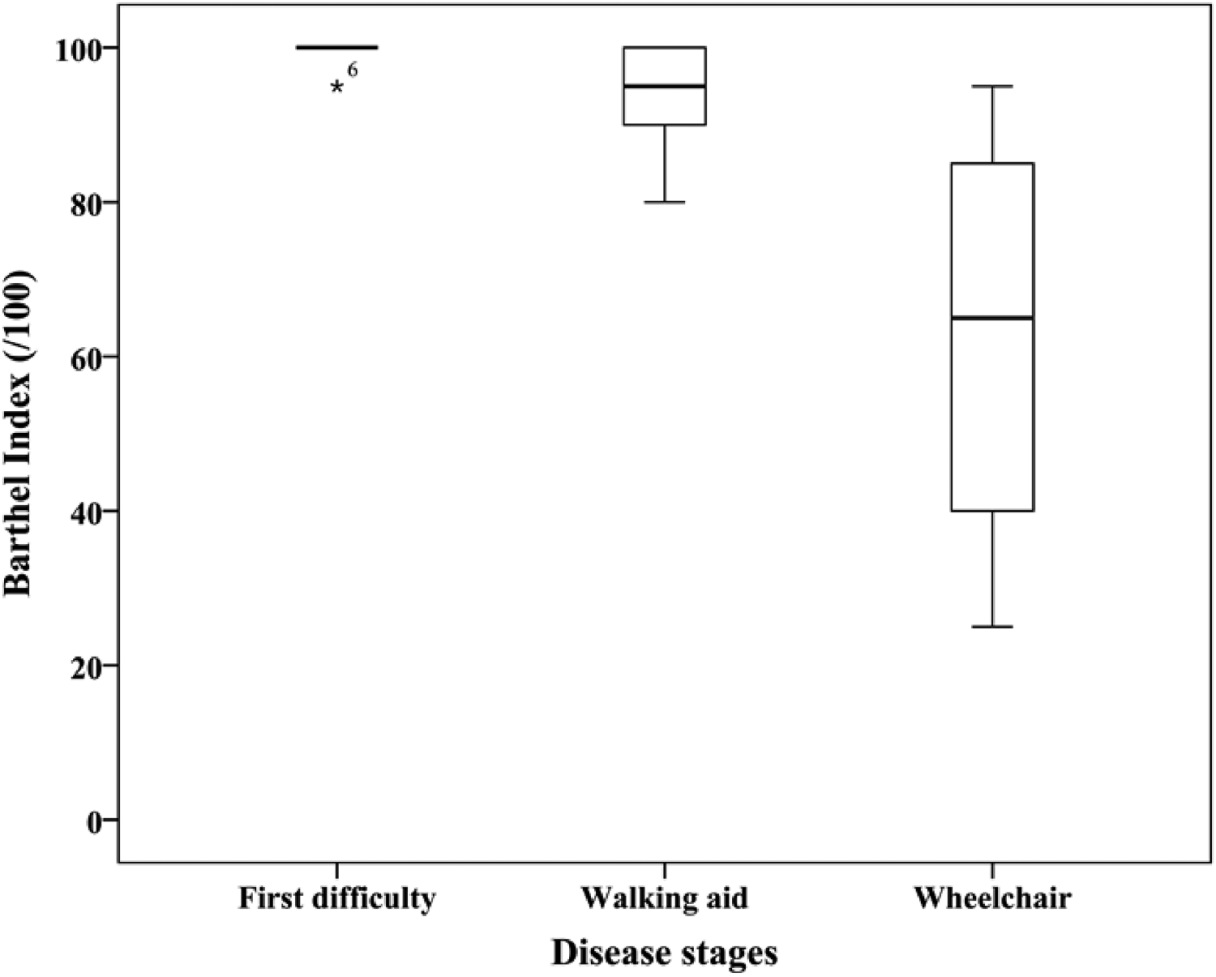
Score distribution of the Barthel Index for each mobility subgroups.

Participants’ satisfaction for each domain of the LIFE-H was also assessed (Supplemental Table 1). The whole group had a median satisfaction of 4.0 out of 5 (First difficulties: 4.7; Walking aid: 4.0; Wheelchair: 4.0). Satisfaction level is statistically different between the three mobility subgroups for only three domains: Communication, Employment, and Leisure. For those domains, participants in the Wheelchair subgroup had a lower level of satisfaction (*p* < .03). Except for Nutrition and Mobility, the accomplishment level correlates (fair to moderate correlations) with the participants’ satisfaction level for all domains, subscores, and total score (*r* = 0.40–0.67, *p* < .03). The total score of the Barthel Index is not normally distributed (*p* < .001), and the results for each mobility level subgroups are shown in Figure 2. No differences between men and women were found. The level of independence in the accomplishment of ADLs is significantly different between the three mobility levels, especially for the Wheelchair subgroup (Table 4 and Figure 2). Items with the lower level of independence are Bladder, Mobility, and Stairs.

**Table 4.** Comparison of Independence in ADLs Between the Three Mobility Level Subgroups as Measured by the Barthel Index

	Independence				*p* value
	Total	First diff. (n = 7)	Walking aid (n = 11)	Wheelchair (n = 10)	
Feeding (/10)	10^a^ [5–10]	10 [10–10]	10 [10–10]	10 [5–10]	.017
Bathing (/5)	5 [0–5]	5 [5–5]	5 [5–5]	2.5 [0–5]	.005
Bowels (/10)	10 [5–10]	10 [10–10]	10 [10–10]	10 [5–10]	.211
Bladder (/10)	10 [0–10]	10 [5–10]	10 [10–10]	5 [5–10]	.198
Mobility (/15)	15 [5–15]	15 [15–15]	15 [10–15]	5 [5–10]	<.001
Stairs (/10)	10 [0–10]	10 [10–10]	10 [10–10]	0 [0–10]	.001
Dressing (/10)	10 [0–10]	10 [10–10]	10 [10–10]	7.5 [0–10]	.005
Grooming (/5)	5 [5–5]	5 [5–5]	5 [5–5]	5 [5–5]	1.0
Toilet use (/10)	10 [0–10]	10 [10–10]	10 [10–10]	10 [0–10]	.018
Transfers (/15)	15 [0–15]	15 [15–15]	15 [15–15]	12.5 [0–15]	.005
Total (/100) [min–max]	95 [25–100]	100 [95–100]	95 [80–100]	65 [25–95]	<.001

^a^ All results are expressed as Median [min–max]; a higher score refers to more independence.

### Associations with Motor Performances

Associations between participation and motor performances are shown in Table 5. All correlations are significant, ranging from moderate to good associations, except for the social activities subscore of the LIFE-H with lower limb coordination and upper limb performance. As for the walking ability, a total of 20 and 19 participants were able to perform the 10mWT and 6MWT, respectively. Participants that were not able to perform these tests were all in the Wheelchair subgroup.

**Table 5.** Association of Participation Level (Measured by the LIFE-H) and Independence (Measured by the Barthel Index) with Motor Performance

			LIFE-H			Barthel Index
			Total score	Daily activities	Social activities	
Lower limb coordination *(n* *=* *28)*			0.62^a^ (<.001)	0.68 (<.001)	0.37 (.055)	0.68 (<.001)
Walking ability	*Comfortable speed* *(n* *=* *20)*		0.71 (<.001)	0.77 (<.001)	0.54 (.015)	0.54 (.015)
	*Long distance* *(n* *=* *19)*		0.72 (<.001)	0.78 (<.001)	0.53 (.018)	0.52 (.018)
Balance *(n* *=* *28)*			0.71 (<.001)	0.81 (<.001)	0.42 (.025)	0.84 (<.001)
Disease severity *(n* *=* *28)*			−0.75 (<.001)	−0.82 (<.001)	−0.48 (.01)	−0.85 (<.001)
Fine dexterity		*NHPT (n* *=* *28)*	−0.62 (<.001)	−0.73 (<.001)	−0.28 (.149)	−0.81 (<.001)
		*PPT (n* *=* *27)*	0.63 (<.001)	0.75 (<.001)	0.26 (.192)	0.85 (<.001)
Upper limb coordination *(n* *=* *28)*			0.41 (.030)	0.54 (.003)	0.095 (.631)	0.64 (<.001)

^a^ Results are expressed as Spearman *r* (*p*-value).

## Discussion

This study highlights the severe impact of ARSACS on participation, which is significantly decreased, even in the intermediate “Walking aid” mobility subgroup, in which participants had a mean age of only 34.7 years. Severely disrupted social participation (activities not accomplished or accomplished with human assistance) was observed, with the most affected domains being Nutrition, Housing, and Mobility. In these domains, most items showed severely disrupted participation from 21% to 80% of our participants. Our results paint a darker picture of participation than the one initially drawn by Gagnon et al. in 2004 (Gagnon, Desrosiers, et al., 2004), and a more restricted participation than in the cohort of healthy elderly participants assessed by Desrosiers, which had a mean age of 73 years (Desrosiers, Bourbonnais, et al., 2005). Considering that a 0.5-point difference in the LIFE-H score is clinically significant (Desrosiers et al., 2006), seven domains are particularly affected in ARSACS as compared to the elderly cohort: Fitness, Personal care, Housing, Mobility, Employment, Community life, and Leisure. For Employment, although no significant differences were found between the three mobility levels, the whole group does not have optimal participation in this social activity. Indeed, the mean score of each indoor mobility subgroups is lower than the score of 7.9 obtained in the healthy elderly group (Desrosiers, Bourbonnais, et al., 2005). The low employment rate in this ARSACS population demonstrates the importance of offering support and guidance regarding work or vocational occupations while considering their long-term abilities and interests, which would be ideal during school years. These long-term decisions require taking several aspects into account in which occupational therapists can definitely bring expertise in regard to long-term prognosis and work evaluation, especially in populations with high clinical variability such as ARSACS (Tremblay et al., 2020). Occupational therapists’ expertise in activity analysis specific to the person's abilities and limitations, and their environment, can contribute to select goals that match his or her current skills level and preferences (Moll et al., 2003). Occupational therapists should also be involved with older adults in finding potential job modifications to facilitate work tasks and in the assessment of residual abilities (Tremblay et al., 2020).

Participation scores also suggest that mobility levels are more closely related to participation in daily activities than social activities. In daily activities, all scores are significantly different between each mobility subgroups. Mobility and Housing are the most affected domains in all three subgroups, and significantly lower scores were recorded for people using a walking aid or wheelchair. These two domains include lower limb coordination and balance demanding tasks, such as getting around on slippery or uneven surfaces or riding a bicycle (Mobility domain) and cleaning or washing the floor (Housing domain), which may explain these results. They are consistent with those obtained with the Barthel Index regarding independence in the accomplishment of ADLs. In fact, four activities were particularly more difficult to achieve: Bathing, Bladder, Mobility, and Stairs. Considering that ARSACS causes great impairments related to mobility and lower limbs motor performance (decreased walking abilities, incoordination, impaired balance) (Gagnon et al., 2018a), these results were not surprising, the same way that restricted participation in the Mobility and Housing domains was expected in the LIFE-H results.

In terms of participation in social activities, three domains out of five show statistically significant scores, including Leisure, where important differences exist between the three mobility subgroups. The use of a walking aid or a wheelchair can restrict access to regular leisure activities. Consideration ought to be given to the accessibility of adapted leisure activities and the impact it may have on this population. Leisure is essential to a satisfying life, especially for people with a disability. It has been found that leisure is positively correlated with life satisfaction and self-esteem, and negatively with depression (Kinney & Coyle, 1992). It is even more relevant in the ARSACS population, since most people do not have a full-time employment and could therefore benefit from a significant hobby that would keep them active and engaged in their community during the day (Bonney et al., 2016). With their client-centered approach, occupational therapists can help find leisure activities that are meaningful to the individual and correspond to his or her level of ability. They can encourage the finding of occupations to maintain feelings of competence and self-determination.

The Mobility, Housing, Leisure, and Community life domains are the most affected in terms of number of participants who reported a severely disrupted participation. Almost all items from these four domains require community mobility. For example, in Community life, half of the items’ wording start with terms such as “Getting to” and “Entering and moving around”. Therefore, it is expected that participants who have a hard time getting out of the house would get lower scores for these items. Stairs, curbs, or sidewalks without cuts, narrow pathways or inaccessible bathrooms are only few examples of environmental barriers that are known to cause restrictions on mobility, independence, and community participation (Evans & McCoy, 1998; Routhier et al., 2019; Stark et al., 2007). In light of these results, rehabilitation teams need to be more involved with the ARSACS population in order to optimize participation either through interventions or the provision of aids, accommodations, or services. Clinical guidelines in ARSACS are lacking but study results in other recessive ataxias could inform best practices to implement. In a study with 42 patients with degenerative cerebellar ataxia, occupational therapy combined with physical therapy led to significantly greater functional gains, more specifically on functional independence, gait, ataxia severity, and falls frequency (Miyai et al., 2012). In this randomized controlled trial, occupational therapy interventions included balance exercises, coordinative tasks of the upper limbs and trunk, and dual motor tasks (Miyai et al., 2012). Interventions in occupational therapy can also involve training of daily activities, with or without the use of assistive devices, modifying the work and home environments, and educating about task simplification and energy conservation techniques (Cup et al., 2008).

Independence in the accomplishment of ADLs (Barthel Index) is also impaired. According to the categories described by Shah et al., the total cohort would be moderately dependent (score 61–90) (Shah et al., 1989). Analysis of these results led us to the same conclusion as Winser et al., which stated that the Barthel Index might not be the most appropriate tool in assessing functional independence in a population with such a chronic condition, partly because of the ceiling effect of the scale (Winser et al., 2017). However, it did allow us to detect significant differences between mobility levels. In fact, according to these same categories, participants in the First walking difficulty subgroup would be almost independent while the Wheelchair subgroup would be at the limit of the severely dependent category (Barthel index total score = 21–60).

For both participation and independence, significant associations were found with motor performance and overall disease severity. If we look at participation, associations are higher in the daily activities subscore than in the social activities subscore of the LIFE-H. Performing daily activities often require motor abilities from either upper or lower limbs, which may be highly affected in this population. A more complete understanding of factors affecting social activities should be undertaken as they could be partially affected by environmental factors such as healthcare and community services. As the disease progresses and motor performance deteriorates, healthcare professionals should pay attention to this situation and suggest interventions to limit their impact on ADLs and help maintaining independence for as long as possible. Since a decreased level of accomplishment is also associated with satisfaction, it is important to address this factor in clinical settings to improve the quality of life of people with ARSACS.

## Study Limitations

This study has some limitations that could limit generalizability of the results. Participation was documented according to mobility level, but each level includes a small number of participants, which could provide an incomplete picture. Moreover, the present study was conducted in a semi-rural area which could hinder generalizability of the findings (i.e., results could be different in a larger city with more accessible public services and transport). In addition, allparticipants were homozygous for the most common mutation of the ARSACS, but some studies have shown that other mutations could lead to a less severe clinical phenotype, which could impact the level of participation in these populations.

## Conclusion

Individuals affected by ARSACS are presenting participation restrictions and decreased independence early on in adulthood. Results also showed an association with motor performances, which suggests that interventions aiming to improve walking capacity or upper and lower limbs functions could help to improve participation and/or independence in this population. This study also highlights specific activities (i.e., housing and mobility) that need to be more carefully monitored by the rehabilitation team to offer care and services in a timely fashion. Further studies with a larger sample size should investigate explanatory factors of participation in the most affected domains in order to offer personalised interventions to individuals living with ARSACS. Finally, qualitative methods would be relevant to get a more in-depth understanding of participation restrictions and perceptions of patients, as done in order rare diseases (Raymond et al., 2016).

## Key Messages

- Daily activities participation restrictions are present as early as mid-adulthood.
- Greater upper and lower limb impairment is more closely related to decreased participation in daily activities than in social activities.
- Occupational therapists can play a key role to assess difficulties in activities such as work and leisure, and consequently offer interventions aimed at offering adaptative solutions.
